# Supplementary material for: High sugar diet–induced fatty acid oxidation potentiates cytokine-dependent cardiac ECM remodeling
Source: J Cell Biol. 2024 Jun 25;223(9):e202306087. doi: 10.1083/jcb.202306087 (PMC11199913; doi:10.1083/jcb.202306087)
Supplement: Table S1 — shows the details of the fly stocks used. [file JCB_202306087_TableS1.docx]

**Table S1: Details of Fly stocks used**

| **Sl** | **Line** | **Genotype** | **Source** |
| --- | --- | --- | --- |
| 1 | *Oregon^R^* | Oregon-R-P2 | BDSC Stock# 2376 |
| 2 | *w^1118^* | *w^1118^* | BDSC Stock# 3605 |
| **Gal4 Driver lines** | | | |
| 1 | *prc-Gal4* | [*w^1118^*](https://flybase.org/reports/FBal0018186.html)*; prc-Gal4* | Chartier et al., 2002 |
| 2 | *dot-Gal4* | [*w^1118^*](https://flybase.org/reports/FBal0018186.html)*; dot-Gal4* | [Kimbrell et al., 2002](https://www.cell.com/cell-reports/fulltext/S2211-1247(14)00143-0?_returnURL=https%3A%2F%2Flinkinghub.elsevier.com%2Fretrieve%2Fpii%2FS2211124714001430%3Fshowall%3Dtrue#bib17) |
| 3 | *yolk-Gal4* | [*w^1118^*](https://flybase.org/reports/FBal0018186.html); *yolk*-Gal4 | Georgel et al., 2001 |
| 4 | *Hnf-Gal4* | *w ^1118^; P{y[+t7.7]w[+mc]=GMR50A12-GAL4}attP2/TM3,Sb[1]* | BDSC Stock # 47618 |
| **UAS lines** | | | |
| 1 | *UAS-bsk^DN^* | *w^1118^, UAS-bsk.DN* | BDSC Stock # 6409 |
| 2 | *UAS-2XEGFP* | *w ^1118^; P{w+MC=UAS-2xEGFP}AH2* | BDSC Stock # 6874 |
| 3 | *UAS-cas9* | [*w^1118^*](https://flybase.org/reports/FBal0018186.html)*;*[*P{GawB}NP5130*](https://flybase.org/reports/FBti0013268.html) [*P{UAS-GFP.U}2*](https://flybase.org/reports/FBti0148305.html)*;* [*P{UAS3xFLAG.dCas9.VPR} attP2*](https://flybase.org/reports/FBti0184669.html) *,* [*P{tubP-GAL80^ts^}2*](https://flybase.org/reports/FBti0027797.html) | BDSC Stock# 67072 |
| 4 | *UAS-mCD8GFP* | *y^*^ w^*^; P{w[+mc]=UAS-mCD8::GFP.L}LL5,P{UAS-mCD8::GFP.L}2* | BDSC Stock# 5137 |
| 5. | *UAS-bmm* | *w*; P{w[+mC]=UAS-bmm.cGa}2* | BDSC Stock# 76600 |
| **Reporter lines** | | | |
| 1 | *upd3-lacZ* | [*w^1118^*](https://flybase.org/reports/FBal0018186.html)*; upd3-lacZ/Cyo* | Zhou et al; 2013 |
| 2 | *TRE-DsRed* | [*w^1118^*](https://flybase.org/reports/FBal0018186.html)*;*[*P{TRE-DsRedT4}attP*](http://flybase.org/reports/FBti0147635.html)*40* | [*BDSC*](http://flybase.org/reports/FBrf0218067.html) *Stock# 59012* |
| 3 | *gstD1-GFP* | [*w^1118^*](https://flybase.org/reports/FBal0018186.html)***;***[*P{GstD1-GFP.S}II*](http://flybase.org/reports/FBti0147635.html) | Sykiotis & Bohmann, 2008 |
| 4 | *Glut1-GFP* | [*y^1^*](https://flybase.org/reports/FBal0018607.html)*w^*^;*[*Mi{PT-GFSTF.0}Glut1^MI02222GFSTF.0^*](https://flybase.org/reports/FBti0178436.html)*/*[*TM6C*](https://flybase.org/reports/FBba0000071.html)*,*[*Sb^1^*](https://flybase.org/reports/FBal0015145.html) [*Tb^1^*](https://flybase.org/reports/FBal0016730.html) | BDSC Stock #59607 |
| 5 | *HexA-GFP* | *w^1118^; PBac{w[+mc]=fTRG00529.sfGFP-TVPTBF} VK00033 FBti0198169* | VDRC Stock# 318587 |
| 6 | *Scully-YFP* | *FlyFos022329(pRedFlp-Hgr)(scu20178::2XTY1-SGFP-V5-preTEV-BLRP-3XFLAG)dFRT* | VDRC Stock# 318761 |
| 7 | *CG3902-YFP* | *w^1118^; PBac{566.P.SVS-1}CG3902^CPTI100004^* | DGRC Stock# 115356 |
| 8. | *pgi-GFP* | *w^1118^; PBac{w[+mc]=Pgii::sfGFP-TVPTBF}attP40 FBtp0142378* | Hudry et al., 2019 |
| **Mutant lines** | | | |
| 1 | *Whd^1^* | *whd^1^* | BDSC Stock #441 |
| 2 | *Mtpα[KO]* | *y^*^ w^*^; TI{w^+*^=TI}Mtpα^KO^ / SM1* | DGRC Stock# 116261 |
| 3 | *Mtpβ[ KO]* | *y^*^ w^*^; TI{w^+*^=TI}Mtpβ^KO^ / SM1* | DGRC Stock# 116262 |
| **UAS RNAi lines** | | | |
| 1 | *UAS-statRNAi* | [*y^1^*](https://flybase.org/reports/FBal0018607.html) [*v^1^*](https://flybase.org/reports/FBal0017656.html)*;*[*UAS-statRNAi^HMS00035^*](https://flybase.org/reports/FBti0140110.html) | BDSC Stock# 33637 |
| 2 | *UAS-domeRNAi* | [*y^1^*](https://flybase.org/reports/FBal0018607.html)*sc^*^*[*v^1^*](https://flybase.org/reports/FBal0017656.html) [*sev^21^*](https://flybase.org/reports/FBal0347491.html)*; UAS-domeRNAi*[*^HMS01293^*](https://flybase.org/reports/FBti0140919.html) | BDSC Stock #34618 |
| 3 | *UAS-upd3RNAi* | [*y^1^*](https://flybase.org/reports/FBal0018607.html)*sc^*^*[*v^1^*](https://flybase.org/reports/FBal0017656.html) [*sev^21^*](https://flybase.org/reports/FBal0347491.html)*; UAS-upd3RNAi*[*^HMS00646^*](https://flybase.org/reports/FBti0140361.html) | BDSC Stock #32859 |
| 4 | *UAS-kayRNAi* | [*y^1^*](https://flybase.org/reports/FBal0018607.html)*sc^*^*[*v^1^*](https://flybase.org/reports/FBal0017656.html) [*sev^21^*](https://flybase.org/reports/FBal0347491.html)*;*[*UAS-kayRNAi^HMS00254^*](https://flybase.org/reports/FBti0140187.html) | BDSC Stock #33379 |
| 5 | *UAS-ask1RNAi* | [*y^1^*](https://flybase.org/reports/FBal0018607.html)*sc^*^*[*v^1^*](https://flybase.org/reports/FBal0017656.html) [*sev^21^*](https://flybase.org/reports/FBal0347491.html)*;*[*UAS-ask1RNAi^HMS00464^*](https://flybase.org/reports/FBti0132158.html) | BDSC Stock #32464 |
| 6 | *UAS-whdRNAi* | [*y^1^*](https://flybase.org/reports/FBal0018607.html)*sc^*^*[*v^1^*](https://flybase.org/reports/FBal0017656.html) [*sev^21^*](https://flybase.org/reports/FBal0347491.html)*;*[*UAS-whdRNAi^HMS00040^*](https://flybase.org/reports/FBti0132158.html) | BDSC Stock #34066 |
| 7 | *UAS-gcn5RNAi* | [*y^1^*](https://flybase.org/reports/FBal0018607.html)*sc^*^*[*v^1^*](https://flybase.org/reports/FBal0017656.html) [*sev^21^*](https://flybase.org/reports/FBal0347491.html)*;*[*UAS-gcn5RNAi^HMS00941^*](https://flybase.org/reports/FBti0132158.html) | BDSC Stock #33981 |
| 8 | *UAS****-****LpR1RNAi* | *y^1^v^1^; UAS-LpR1RNAi*[*^JF02551^*](https://flybase.org/reports/FBal0239254.html) | BDSC stock # 27249 |
| 9 | *UAS****-****LpR2RNAi* | *y^1^ sc^*^ v^1^ sev^21^; UAS-LpR2RNAi^HMS03722^* | BDSC stock # 54461 |
| 10 | *UAS-AcCoASRNAi* | *y^1^ sc^*^ v^1^ sev^21^; UAS-AcCoASRNAi^HMS02314^* | BDSC stock # 41917 |
| 11 | *UAS-chmRNAi* | *y^1^v^1^; UAS-chmRNAi*[*^JF02348^*](https://flybase.org/reports/FBal0239254.html) | BDSC stock # 27027 |
| **Crisper lines** | | | |
| 1 | *whd* *^TKO.GS00854^* | [*y^1^*](http://flybase.org/reports/FBal0018607.html)*sc^*^*[*v^1^*](http://flybase.org/reports/FBal0017656.html) [*sev^21^*](http://flybase.org/reports/FBal0347491.html)*;*[*whd^TKO.GS00854^*](http://flybase.org/reports/FBti0195046.html) | BDSC Stock #77066 |
| 2 | *whd ^TOE.GS005326^* | [*y^1^*](http://flybase.org/reports/FBal0018607.html)*sc^*^*[*v^1^*](http://flybase.org/reports/FBal0017656.html) [*sev^21^*](http://flybase.org/reports/FBal0347491.html)*; whd^TOE.GS00536^* | BDSC Stock #68139 |
