# Supplementary material for: High sugar diet–induced fatty acid oxidation potentiates cytokine-dependent cardiac ECM remodeling
Source: J Cell Biol. 2024 Jun 25;223(9):e202306087. doi: 10.1083/jcb.202306087 (PMC11199913; doi:10.1083/jcb.202306087)
Supplement: Table S2 — lists primers used. [file JCB_202306087_TableS2.docx]

**Table S2: List of Primers Used**

| **Sr. No.** | **Gene Name** | **Primer Sequence (Forward and Reverse)** |
| --- | --- | --- |
| 1 | *Prc* | AGAGGCTATTCGAGGGGACAA  GGAGCGAGATCCATTTTCGGTA |
| 2 | *Upd3* | AGCCGGAGCGGTAACAAAA  CGAGTAAGATCAGTGACCAGTTC |
| 3 | *rp49* | CTAAGCTGTCGCACAAATGGC  TTCTGCATGAGCAGGACCTC |
| 7 | *Dilp2* | AGGAACGCTCTGCAGTGAAA  CGGCACCGGGCATGG |
| 8 | *Dilp3* | AGCACCTACAGAAGCGTTCC  TGCAGGATCCTTTGGCGTTT |
| 9 | *Dilp5* | GCCTTGATGGACATGCTGA  AGCTATCCAAATCCGCCA |
| 10 | *Whd* | GGCCAATGTGATTTCCCTGC  TGCCCTGAACCATGATAGGC |
| 11 | *Scully* | GATCAAGAACGCCGTTTCCC  CAGATCGGCCAGGATCACG |
| 12 | *Mtpα* | ATCACTGTTGGTGACGGACC  CTGCAGCAGTCTGATGGCTT |
| 13 | *Mtpβ* | CAGGCACTCGCTTTTGTCAT  CCTGGCAATGTTGGAGGTCT |
| 14 | *Yip2* | TCTGCCGCAACCAAAGGTAT  TTAAGACCGGCAGCATCCAG |
| 15 | *Mcad* | GGCCTGGATCTCGATGTGTT  GATCACAGGAGTTTGGCCCAG |
| 16 | *Gcn5* | CGATCGTCCAAGCAGTGAGA  TGGAACTCAATGGCTCGCTT |
| 17 | *Chm* | CGATGTGGAGCCCTTCCTTT  TGCAGAATGGTTGTAAATGAAATCG |
| 18 | *Gpat4* | CTACCCGGTGGCCATTAAGT  TTCGCGATGACACTCTTCAC |
| 19 | *Agpat4* | CACGGTTCTTTCCATTTTGG  CGTATATTCCAAAACTGTTGCAG |
| 20 | *Dgat2* | TGTCCAAGTTGTTGGTGCTC  GGCACTCTTCGAATTCTCCA |
| 21 | *Bmm* | AATGCCCACGACTCCTGAAC  TCGATGAAGAAGTCTGCGCC |
| 22 | *Hsl* | GCAACGTGATGGCTCAACAG  AGGGGTCCATGTTAAGTGTAAGT |
| 23 | *Acc* | GTGCAACTGTTGGCAGATCAGTA  TTTCTGATGACGACGCTGGAT |
| 24 | *Fasn1* | CCCCAGGAGGTGAACTCTATC  GACTTGACCGATCCGATCAAC |
