# Supplementary figures and images for: High sugar diet–induced fatty acid oxidation potentiates cytokine-dependent cardiac ECM remodeling
Source: J Cell Biol. 2024 Jun 25;223(9):e202306087. doi: 10.1083/jcb.202306087 (PMC11199913; doi:10.1083/jcb.202306087)

# Insulin (-)

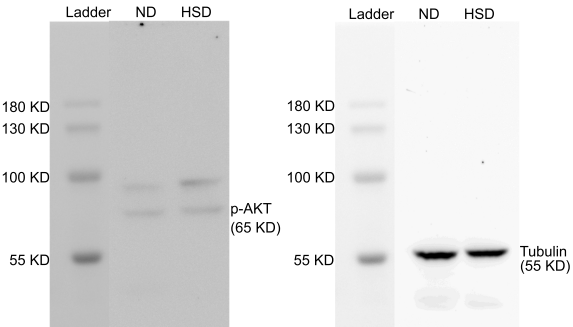

# Insulin (+)

Ladder ND HSD

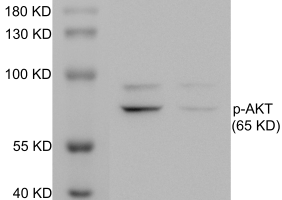

Ladder ND HSD

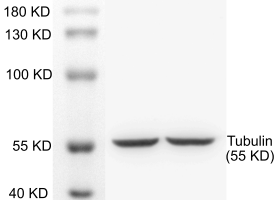

Supplement: SourceData FS1 — is the source file for Fig. S1. [file JCB_202306087_SourceDataFS1.pdf]
